# Regional differences in the effects of the ablation index and interlesion distance on acute electrical reconnections after pulmonary vein isolation

**DOI:** 10.1002/joa3.12397

**Published:** 2020-07-16

**Authors:** Kyoichiro Yazaki, Koichiro Ejima, Satoshi Higuchi, Daigo Yagishita, Morio Shoda, Nobuhisa Hagiwara

**Affiliations:** ^1^ Department of Cardiology Tokyo Women’s Medical University Shinjuku‐ku Japan; ^2^ Clinical Research Division for Heart Rhythm Management Department of Cardiology Tokyo Women’s Medical University School of Medicine Shinjuku‐ku Japan

**Keywords:** ablation index, acute pulmonary vein‐left atrium reconnection, interlesion distance, left posterior segments, pulmonary vein isolation

## Abstract

**Background:**

In pulmonary vein isolation, the regional differences in the ablation index (AI) and interlesion distance (ILD) remain unclear. This study aimed to evaluate the association between the AI, ILD, and other relevant indices with pulmonary vein reconnections (PVRs) during the surgical intervention with a focus on the heterogeneous regional variability through a retrospective analysis.

**Methods:**

We divided the wide area circumferential ablation (WACA) region into 12 segments in 32 consecutive patients, which resulted in a 384 segment analysis to evaluate the association of the minimum AI (AI min) and maximum ILD (ILD max) with acute PVRs, which were defined as spontaneous PVRs or dormant conduction after adenosine triphosphate administration.

**Results:**

Acute PVRs were observed in 48 (13%) segments and 40 (63%) WACA regions. The AI min was significantly lower and ILD max greater in segments with PVRs than in those without (372 vs 403 au and 6.5 vs 5.7 mm, respectively). PVRs were more frequent in the left posterior segments, adjacent to the esophagus, than in other segments (23% vs 10%, respectively). Notably, ILD max was significantly greater in the left posterior segments with acute PVRs with AI min < 297 (median; 6.5 vs 5.1 mm); a similar finding was not observed when with AI min ≥ 297.

**Conclusion:**

Smaller ILD may prevent acute PVRs when the AI min is low in the left posterior segments.

## 1 INTRODUCTION

For approximately two decades, pulmonary vein isolation (PVI) has been developed as an effective therapeutic strategy of paroxysmal atrial fibrillation (AF) refractory to anti‐arrhythmic drugs.^1^ The two major determining factors of durable PVI are as follows: (a) transmural lesions, derived from several factors, including contact force (CF), radiofrequency (RF) time, and delivered energy, which have recently been integrated into a specific formula to calculate the ablation index (AI)^2^ and (b) the interlesion distance (ILD), which reflects lesion contiguity visualized by the tags on three‐dimensional (3D) electroanatomical mapping.^3^ Nevertheless, pulmonary vein (PV)‐left atrium (LA) reconnections (PVRs) are observed in some patient populations and are substantially associated with underlying anatomical factors, such as wall‐thickness variability in the LA.^4^ Although studies have provided evidence of an association between AI or ILD and PVRs,^2^, ^5^ a detailed evaluation focusing on regional differences in the AI and ILD has not been conducted. Therefore, we aimed to evaluate the association of the AI, ILD, and other relevant indices with PVRs during the procedure with a focus on the heterogeneous regional variability through a retrospective analysis.

## 2 METHODS

### 2.1 Study population

A total of 32 patients who underwent RF catheter ablation for AF for the first time between April 16 and June 27 in 2018 at the Tokyo Women's Medical University Hospital were included. All anti‐arrhythmic drugs were stopped for at least five half‐lives. None of the patients had a history of amiodarone use or implantable cardiac devices. All patients provided written informed consent. The study protocol conformed to the ethical guidelines of the 1975 Declaration of Helsinki and was approved by the institutional review board and ethical committee of Tokyo Women's Medical University Hospital.

### 2.2 Catheter ablation protocol

Prior to the ablation sessions, 3D cardiac computed tomography was performed within a week prior to the procedure to detect the location of the esophagus, understand the geometric features of the LA, and rule out any thrombus in the LA. Transthoracic echocardiography was also performed within a month prior to the procedure, and transesophageal echocardiography was performed in selected patients, such as those with a high CHADS2 score or longstanding AF. The details of the catheter ablation protocol have been described previously.^6^ Briefly, we first inserted a multisensor probe (Esophastar, Japan Lifeline) nasally to monitor the esophageal temperature and detect the location of the esophagus, and the alert was set at an upper temperature limit of 39°C. Subsequently, all patients were deeply sedated with a 10‐minute continuous administration of dexmedetomidine at 6 µg/kg/h, followed by a continuous infusion of 0.3‐0.7 µg/kg/h. Under uninterrupted oral anticoagulation, 5000 IU of heparin was administered after inserting three sheaths (one short and two long). A decapolar‐electrode catheter was positioned in the coronary sinus, and a transseptal puncture under intracardiac echocardiography guidance was performed, resulting in the introduction of two long sheaths into LA. All patients underwent wide circumferential PVI guided by electroanatomical mapping combined with geometric information with reconstructed computed tomography images using a 3D mapping system (CARTO 3, Biosense Webster, Inc). All RF applications (RFA) were performed in a point‐by‐point fashion, avoiding perpendicular catheter contact as possible. In case of persistent AF, electrical cardioversion was performed prior to RFA. We used a single circular catheter as a mapping catheter and a 3.5‐mm open‐irrigated tipped catheter (ThermoCool STSF, Biosense Webster, Inc) as the ablation catheter using power of 25‐40 W with an upper limit temperature of 42°C. The power was limited to 20‐25 W, and the upper limit for the RF time was 15 seconds at the posterior wall adjacent to the esophagus in the LA to avoid any injury. According to a previous study, the attenuation of the negative deflections in the unipolar electrogram recorded at the distal tip of the ablation catheter was an indicator of sufficient transmural necrosis.^7^ We terminated the RFA 5‐10 seconds after the elimination of the negative component of the unipolar electrogram. When a change in the unipolar electrogram was not observed, RFA was continued for up to 30 seconds (15 seconds in the posterior segments adjacent to the esophagus) or the RF power was increased up to 40 W (other than the posterior segments). To clearly visualize the unipolar signals, we introduced a 10‐polar electrode catheter with an indifferent electrode (DECANAV^®^, Biosense Webster, Inc) into the coronary sinus. The catheter tip was irrigated with saline at a flow rate of 2 mL/min during mapping and 8‐15 mL/min during ablation. Superior vena cava isolation was performed after PVI. Subsequently, induction of AF was performed by atrial overdrive pacing with a 10‐μg isoproterenol infusion. If PVRs were observed after that, re‐isolation was performed using the ablation catheter in the guidance of the earliest activation site in the circular mapping catheter. Once entrance block was achieved after RFA, we performed pacing (5 mA, 1 ms) within the isolation line from the ablation or circular mapping catheter to confirm exit block. Subsequently, adenosine triphosphate (ATP) was intravenously injected to confirm the absence of any PV‐LA dormant conduction (DC) with the ring and ablation catheters in the right and left PVs, respectively. The confirmation was performed for all PVs using a two‐by‐two method for a minimum of 20 minutes after the isolation of the ipsilateral PV pair; specifically, once DC was observed, the electrically earliest site was detected by inserting the circular catheter in the target PV. We subsequently sought to identify the gap site as close to the isolation line as possible using the ablation catheter. In case DC was transient, we sought to detect the most possible reconnection sites using the guidance of potentials recorded by the circular mapping catheter using a repeated injection of ATP. After each RFA at the possible site, we confirmed the absence of DC using ATP to accurately determine the reconnection sites. This cycle was repeated until DC was eliminated. No further waiting period was applied following re‐isolation or re‐elimination of DC.

### 2.3 Ablation index and interlesion distance measurement

Each wide area circumferential ablation (WACA) region was divided into six segments consisting of two anterior, two posterior, roof, and inferior segments, resulting in a total of 12 segments per patient. We defined acute PV reconnections (PVRs) as spontaneous PV‐LA reconnections or DC, where the touch‐up applications achieved sequence changes at the circular catheter, or completed the bidirectional block of isolation lines, and measured several indices, including AI and ILD at each segment. During the procedure, each parameter, including the delivered power, impedance drops, CF (g), force‐time integral (FTI), and AI were measured on‐site. AI is an indirect index for predicting the lesion transmurality based on an established formula,^2^ which includes CF, RF time, and delivered power. The ILD was the center‐to‐center distance between neighboring ablation tags. These parameters were measured per segment and visualized by the operators during the procedure; there was no blinding during the procedure. We sought to perform RFA during the normal phase of breathing (not during deep inspirations or when breathing frequently) even after respiratory adjustment to accurately obtain the 3D mapping‐related indices. Automated ablation tagging (VisiTag, Biosense Webster, Inc) was used to mark the place of each lesion. VisiTag was set as follows: for stability, the catheter was positioned within 2.5‐mm for at least 3 s with a force over time of 25%, minimum gravity force of 3 g, tag size of 2 mm, and white‐colored tags were assigned when the AI was less than 320 au and red‐colored tags when the AI was more than 380 au. All VisiTags were annotated with the segment name (eg, left postero‐inferior segment) during the procedure on‐site; furthermore, we manually tagged the acute PVR sites where re‐isolation or a sequence change in PV was observed during the procedure, which were counted retrospectively. We determined the PVR or DC sites according to the neighboring VisiTags at the first encircling to define each segment; therefore, the colors of the VisiTags were not completely blinded to us at the time of determining the acute PVR site. Nevertheless, because the PVR site was approximately equal to the site where re‐isolation or sequence change was observed in the touch‐up procedure, the VisiTag color could not affect our decision for the PVR site. Furthermore, 3D mapping‐related indices were entirely blinded to us during the analysis. If VisiTags overlapped, both tags were included in the analysis of RF time or energy but were individually analyzed when considering 3D mapping‐related indices (eg, lower value in AI min and larger value in ILD max were used) to increase the accuracy.

### 2.4 Statistical analysis

Categorical variables are expressed as mean ± standard deviation or median with interquartile range. Student's *t* test and Wilcoxon test were used to compare continuous variables between the groups. Fisher's exact test and Kruskal‐Wallis test were used to evaluate differences between categorical and continuous variables among the segments of WACA for the analysis of variance. Kaplan‐Meier curve analysis was used to assess atrial tachyarrhythmia recurrence‐free survival rate after the single procedure. All findings were considered statistically significant at a *P* < .05. All statistical analyses were performed with JMP^®^ 13 software (SAS Institute Inc).

## 3 RESULTS

The baseline characteristics are summarized in Table 1. A total of 32 patients were analyzed. The mean age was 65 ± 11 years, 18 (62%) patients were male, and 29 (91%) had paroxysmal AF. The mean left atrial dimension and left ventricular ejection fraction were 39 ± 8 mm and 56 ± 7%, respectively. No anomalous PVs, including right middle branch or left common PV, were observed. We ensured that the esophagus was located on the left of the LA in all patients using cardiac computed tomography. First‐pass isolation was achieved in 88% and 63% of patients in right and left PVs, respectively, and all PVs were successfully isolated.

**TABLE 1:** Patient characteristics

Patient characteristics	
Mean age, y	65 ± 11
Male	18 (62)
Paroxysmal AF	29 (91)
Structural heart disease	7 (24)
Coronary artery disease	1
Cardiomyopathy	4
Hypertensive heart disease	1
Other	1
Right‐sided esophagus	0 (0)
Echocardiographic parameter	
LAD, mm	39 ± 8
LAV, mL	67 ± 22
LAVI, mL/cm^2^	41 ± 15
LVEF, %	56 ± 7
Time to complete WACA	
Right side, min	19 ± 11
Left side, min	28 ± 14
Total procedure time, min	150 ± 50
Acute PVR	29 (90)
RF application at the carina	
Right side	3 (9)
Left side	1 (3)

Note

Values are expressed as n (%), or mean ± SD.

Abbreviations: AF, atrial fibrillation; LAD, left atrial diameter; LAV, left atrial volume; LAVI, left atrial volume index; LVEF, left ventricular ejection fraction; PVR, pulmonary vein reconnection; RF, radiofrequency; WACA, wide area circumferential ablation.

Acute PVRs were observed in 48 (13%) segments and in 40 (63%) WACA regions. The acute PVR segments consisted of 43 segments with spontaneous PVRs (11%), six with DC (2%), and one with both (0.2%). The observed values for RF time, CF, power, decrease in impedance, number of tags, FTI, AI min, and ILD max were 18 (12‐22) minutes, 9 (7‐12) g, 30 (25‐35) W, 4.8 (3.2‐7.0) ohm, 5 (3‐6), 190 (131‐263) gs, 400 (331‐444) au, and 5.8 (5.2‐7) mm per segment, respectively. In a total of 72 (19%) segments, an ILD max less than 5 mm was achieved. All spontaneous PVRs and DC were successfully treated with touch‐up RFA during the procedure.

### 3.1 Segments with acute PVRs vs those with no PVRs

**FIGURE 1:**
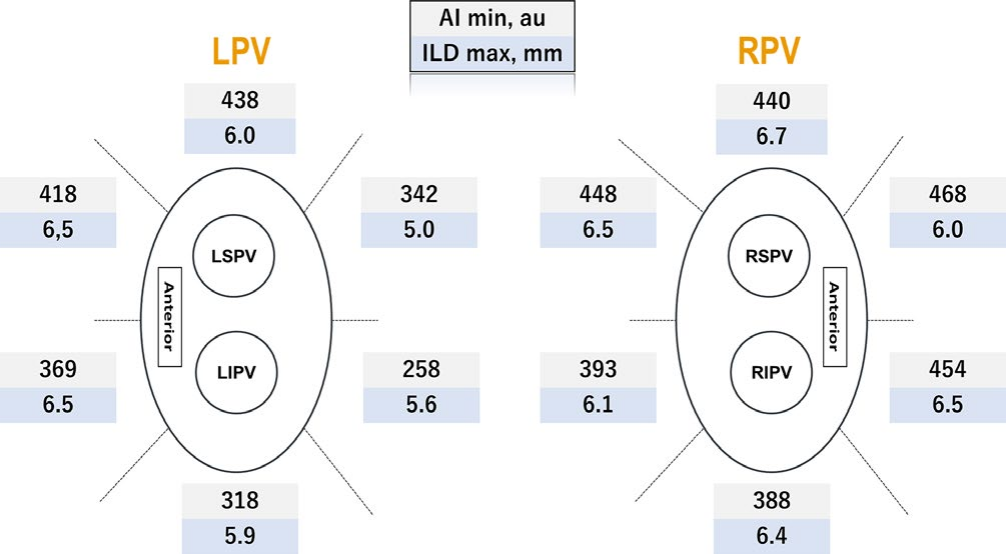
Differences in the minimum ablation index (AI min) and maximum interlesion distance (ILD max) between the segments. AI min, minimum ablation index; ILD max, maximum interlesion distance; LIPV, left inferior pulmonary vein; LPV, left pulmonary vein; LSPV, left superior pulmonary vein; RIPV, right inferior pulmonary vein; RPV, right pulmonary vein; RSPV, right superior pulmonary vein

**FIGURE 2:**
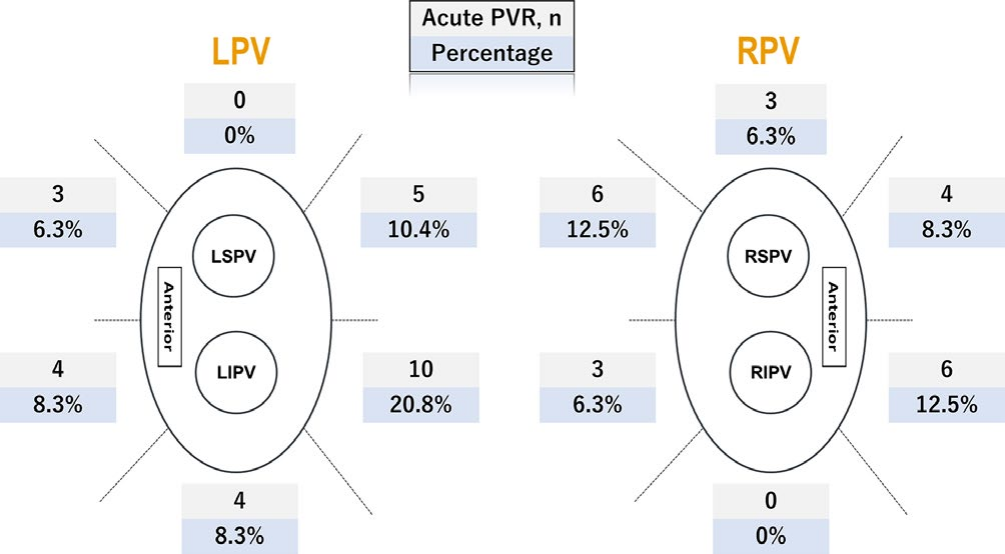
Differences in the number of acute PVRs and the occurrence rate between the segments. LIPV, left inferior pulmonary vein; LPV, left pulmonary vein; LSPV, left superior pulmonary vein; PVR, pulmonary vein reconnection; RIPV, right inferior pulmonary vein; RPV, right pulmonary vein; RSPV, right superior pulmonary vein

AI min and ILD max were heterogeneously distributed among the 12 segments (*P* < .0001 and *P* < .0001, respectively; Figure 1). The frequency of acute PVR also significantly differed between the segments (*P* = .01; Figure 2). In the segments with acute PVRs, AI min was significantly lower than that in segments without acute PVRs [372 (301‐416) vs 403 (339‐446), respectively, *P* = .01]. However, in the per‐segment analysis, there was no significant difference in the AI min between the segments with PVRs and those without, except in the right supero‐posterior segment (391 [354‐406] vs 429 [405‐479], respectively, *P* = .02; Table 2). Likewise, ILD max demonstrated a significant difference between the segments with acute PVRs and those without (6.5 [5.5‐7.3] vs 5.7 [5.1‐6.9], respectively, *P* = .01). In regional assessments, ILD max was significantly higher in the reconnected sites on the left infero‐posterior wall (6.4 [5.9‐6.6] vs 5.3 [4.8‐5.6], *P* = .006), while no significant difference was observed in the other segments. For the other parameters, there was a significant difference in the number of tags, RF time, decrease in impedance, and FTI between the segments with PVRs and those without (Table 3). A rise in the esophageal temperature interrupted RF application in 49 of 64 (77%) left posterior segments (32 left supero‐posterior and 32 left infero‐posterior). There was no significant difference in the rate of increase of esophageal temperature between segments with acute PVR and those without in the left posterior segments (87% vs 73%, *P* = .49).

**TABLE 2:** Comparison of the minimum ablation index between the segments with acute pulmonary vein reconnections and the other segments: regional differences

Segment	Right		*P*‐value
	Acute PVR	No acute PVR	
	(n = 22)	(n = 170)	
Roof	434 (365‐519)	445 (389‐472)	.95
Ant			
Superior	412 (392‐459)	449 (437‐491)	.06
Inferior	447 (421‐463)	454 (406‐484)	.54
Post			
Superior	391 (354‐406)	429 (405‐479)	.02
Inferior	385 (364‐459)	393 (368‐424)	.95
Inf	—	388 (349‐413)	—

Segment	Left		*P*‐value
	Acute PVR	No acute PVR	
	(n = 26)	(n = 66)	
Roof	—	438 (382‐478)	—
Ant			
Superior	415 (395‐425)	419 (375‐438)	.70
Inferior	321 (251‐382)	371 (327‐419)	.12
Post			
Superior	339 (307‐432)	343 (296‐366)	.53
Inferior	257 (222‐279)	258 (236‐303)	.61
Inf	305 (227‐334)	321 (285‐340)	.38

Note

Values are presented as median (interquartile range).

Abbreviations: Ant, anterior; Inf, inferior; Post, posterior; PVR, pulmonary vein reconnection.

**TABLE 3:** Comparison of the relevant parameters between the segments with acute pulmonary vein reconnections and other segments

Variables	Acute PVR	No acute PVR	*P*‐value
	(n = 48)	(n = 336)	
The number of tags, n	5 (4‐8)	4 (3‐6)	.03
RF time, s	16 (9‐21)	18 (13‐22)	.049
RF power, W	30 (25‐35)	30 (25‐35)	.23
CF, g	8.7 (6.8‐11.6)	8.8 (6.7‐12.3)	.82
FTI, gs	385 (364‐459)	393 (368‐424)	.02
Impedance decrease, ohm	4.1 (2.9‐5.8)	4.9 (3.5‐7.4)	.007

Note

Values are presented as median (interquartile range).

Abbreviations: CF, contact force; FTI, force‐time integral; PVR, pulmonary vein reconnection; RF, radiofrequency.

### 3.2 Comparison of the posterior segments with the other segments

AI min was significantly lower in the left posterior segments than in other segments (297 [253‐344] vs 412 [356‐450], respectively, *P* < .0001). Furthermore, more acute PVRs were observed in the left posterior segments than in the other segments (23% vs 10%, respectively, *P* = .007).

**FIGURE 3:**
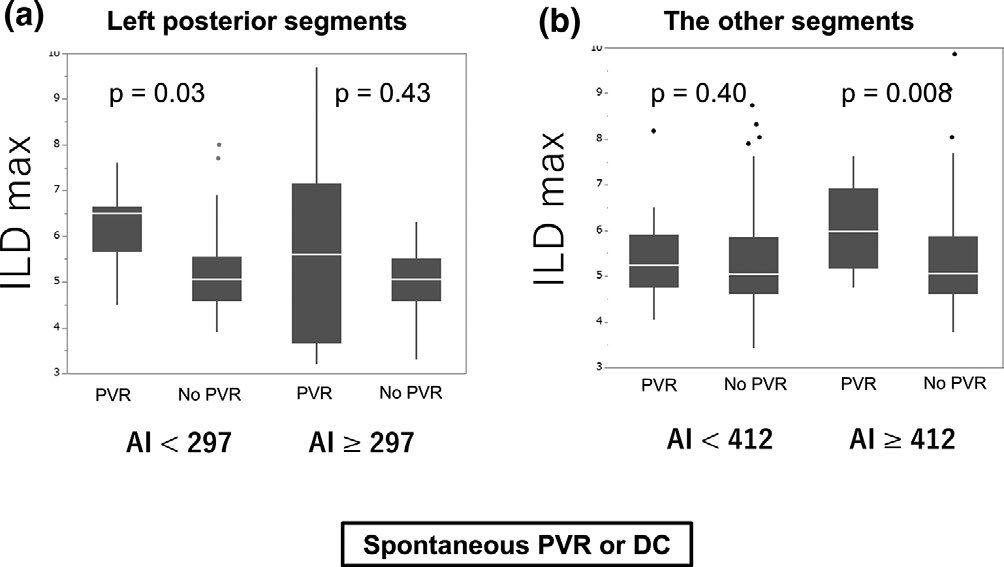
Panel A illustrates the comparison of the maximum interlesion distance (ILD max) between the segments with acute pulmonary vein reconnections (PVRs) and the other segments according to the minimum ablation index (AI min) value in the left posterior segments; the segments with AI min lower than the median AI min and higher than the median AI are indicated on the left and right sides, respectively. Panel B illustrates the same for the other segments. AI min, minimum ablation index; DC, dormant conduction; PVR, pulmonary vein reconnection

Table 4 summarizes the differences in ILD max between the segments with acute PVRs and those without, which were categorized by the median AI value in the posterior (left, right, and both) and other‐than‐posterior segments. When we divided the left posterior segments into two groups according to the median AI min value (297), acute PVRs were observed in nine (28%) segments in nine patients and were not observed in 23 (72%) segments in 20 patients in the low AI min (<297) group. In this subgroup, ILD max was significantly higher in the segments with acute PVRs than that in those without (6.5 [5.7‐6.7] vs 5.1 [4.6‐5.6], respectively, *P* = .03; Figure 3A), while this was not observed in the high AI min group. Furthermore, in the left posterior segments, CF, FTI, and decrease in impedance demonstrated no significant difference between these two groups (8.5 g [6.8‐1.7] vs 8.9 g [7.1‐14.1], *P* = .59; 70 gs [55‐107] vs 85 gs [67‐119], *P* = .42; 3.8 ohm [3.2‐5.0] vs 4.2 ohm [1.5‐5.2], *P* = .73, respectively).

**TABLE 4:** Differences in ILD max according to the presence of acute PVR when categorized based on the AI min value and regions

Segment	AI min < median			AI min ≥ median		
	PVR (+)	PVR (−)	*P*‐value	PVR (+)	PVR (−)	*P*‐value
Left posterior	6.5 (5.7‐6.7)	5.1 (4.6‐5.6)	.03	5.6 (3.7‐7.2)	5.1 (4.6‐5.5)	.4
Right posterior	6.6 (5.6‐7.1)	5.8 (5.2‐6.4)	.1	8 (7.2‐8.8)	5.8 (5.3‐7.2)	.28
Posterior	6.3 (5.5‐6.6)	5.2 (4.6‐5.8)	.02	6.6 (5.6‐8)	5.7 (5.2‐6.8)	.12
Other‐than posterior	6.2 (5.4‐7.0)	6.0 (5.3‐7.1)	.91	7.2 (6‐8)	6.0 (5.3‐7.0)	.02

Note

Values are presented as median (interquartile range).

Abbreviations: AI, Ablation index; PVR, pulmonary vein reconnection.

In contrast, in the right posterior segments, acute PVRs were observed in two (6%) segments in two patients and were not observed in 30 (94%) segments in 22 patients when AI min was low (<407). ILD max demonstrated no significant difference between the segments with acute PVRs and those without when AI min was low (<407) (6.6 [5.6‐7.1] vs 5.8 [5.2‐6.4], respectively, *P* = .10). A similar observation was noted in the same subgroup when AI min was high (≥407).

In all posterior segments, acute PVRs were observed in 15 (24%) segments in 15 patients and were not observed in 47 (76%) segments in 26 patients when AI min was low (<364). ILD max demonstrated a significantly higher value in segments with acute PVRs than in those without when AI min was low (6.3 [5.5‐6.6] vs 5.2 [4.6‐5.8], respectively, *P* = .02); however, this was not observed when AI min was high.

In the other‐than‐posterior segments, acute PVRs were observed in 11 (9%) segments in 11 patients and were not observed in 116 (91%) segments in 32 patients when AI min was high (≥415), and ILD max demonstrated a significantly higher value in segments with acute PVRs than in those without (7.2 [6.0‐8.0] vs 6.0 [5.3‐7.0], respectively, *P* = .02). This was not observed when AI min was low. Therefore, ILD max was significantly higher in the segments with acute PVRs than in those without when AI min was high (≥412) (7.3 [6.1‐8.6] vs 6.0 [5.3‐7.1], respectively, *P* = .008), while this was not observed when AI min was low (<412) (6.2 [5.5‐7.2] vs 5.9 [5.3‐7.1], respectively, *P* = .40) in the other‐than‐left‐posterior segments (Figure 3B). When the outcome included only spontaneous PVR, similar findings were noted (Figure S1).

### 3.3 Follow‐up

During a median follow‐up of 21 months, 11 patients experienced atrial tachyarrhythmia recurrence with a cumulative atrial tachyarrhythmia‐free survival rate of 65% at 1 year after the first procedure. Excluding patients with structural heart disease, this rate increased to 76% at 1 year. No late complication occurred during the follow‐up period. Three patients underwent redo procedure after the blanking period, during which late PVR was observed in all cases. The late PVR site was the same as the acute PVR site of the previous session in only one case (left posterior segments; carina portion).

## 4 DISCUSSION

In this study, we identified a substantial contribution of AI min and ILD max to the incidence of acute PVRs in a whole‐segment analysis. Additionally, we demonstrated the importance of ILD max in the incidence of acute PVRs in segments with low AI min (<297) among the left posterior segments and of those with high AI min (≥412) among the other‐than‐left‐posterior segments.

Acute reconnections that included ATP‐induced DC and spontaneous reconnections with/without isoproterenol infusion were observed in 13% of the segments. These results suggest the difficulty in achieving ipsilateral isolation lines without acute PVRs. A previous meta‐analysis revealed that adenosine‐guided PVI improved the arrhythmia‐free survival rate more than conventional PVI,^8^ and a previous study reported that isoproterenol infusion and waiting time also facilitated acute PVRs. However, acute reconnections were considered not to be associated with late recurrences if they were appropriately treated. One possible reason for these findings was acute edema, which contributed to reversible lesion creation. Nevertheless, we believe that these findings should not encourage us to disregard achieving the absence of acute PVR. Clearly, a longer procedure time, greater RFA energy, longer RFA time, and greater radiation exposure were required when touch‐up application was needed, all of which were harmful not only for the patients but also for the operators. Furthermore, a recent study reported that longer RF time was required in patients with acute PVR.^9^ Therefore, we should seek to explore the optimal procedure to achieve the absence of acute PVR, including first‐pass isolation, which has been defined as isolation by one‐time encircling.

We demonstrated that a high AI min was associated with a low incidence of acute PVR. In the FTI formula, CF is multiplied by the delivery time; this results in a linear relationship between the FTI and CF or time. The AI is a more sophisticated indicator of the lesion transmurality; it weights the delivered power in addition to the CF and RFA times and demonstrates a nonlinear relationship with the lesion dimension, unlike the FTI. Therefore, compared with the FTI, the AI is a more acceptable predictor of PVRs and provides better atrial tachyarrhythmia‐free survival rates^10^ thanks to the precise estimation of the lesion width and depth.^11^

AF recurrence is also associated with lesion contiguity. El Haddad et al reported that AI and ILD were independently associated with PVR in the CF‐guided PVI procedure.^12^ Park et al reported that PVRs are associated with ILD, and when considering sites with high CF with PVR, ILD was more than 6.6 mm.^3^ We also found significant differences in ILD max according to the presence of PVR. However, this difference disappeared in the per‐segment analysis, possibly because of the small number of tags in each segment. Furthermore, in approximately 80% of the segments, ILD ≥ 5 mm was observed in at least one pair of tags, which was partially associated with this result because the difference in ILD max faded out in the regional assessment. This is partially because the target ILD was not sufficiently achieved, which resulted in the variability of the ILD max.

In the per‐segment analysis, there was scattering of AI min value and a small statistical difference was observed. This was because of the geometrical features of LA and strategical differences. As broadly understood, the posterior segments have the following specific features: thinner walls than the anterior segments and the segments of the area adjacent to the esophagus mainly on the left side. These features have a significant impact on outcomes following RFA, that is, insufficient RFA was frequently observed. Several studies have reported differences in the wall thickness of LA. Beinart et al reported an average LA wall thickness of 1.89 mm in the posterior wall, and the mid‐superior aspect was the thinnest, as measured by computed tomography.^4^ Platonov et al performed an autopsy study of patients with history of AF and found a significant difference in the LA wall thickness, especially on the left posterior wall in the central and inferior levels.^13^ In this regard, the AI min was targeted at more than 300 au in the area adjacent to the esophagus when increasing esophagus temperatures were observed to avoid LA esophagus fistulas.^12^ In the present study, acute PVRs were more frequently observed in the left posterior segments than in the other segments, which accounted for more than 30% of all PVRs. This guided us to improve the outcomes in this region by considering the regional specificity. Our data revealed that AI min was significantly smaller in the left posterior segments than in the other segments, primarily because temperature increases in the esophagus that might be associated with acute PVR were observed more frequently in the left posterior segments than in the other segments. Furthermore, we demonstrated significant differences in ILD max between the segments with acute PVR and those without in the left posterior segments when AI min was low, suggesting that the ILD max played a crucial role in the creation of durable lesions when sufficient CF, power, or RF time was not applied.

**FIGURE 4:**
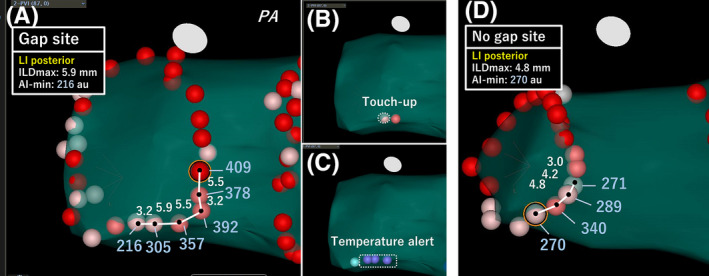
Example of cases with and without a gap site in the left posterior segment. (A) A case with a gap site in the left infero‐posterior segments showing six VisiTags, white and blue numbers indicate the center‐to‐center (black dot‐to‐black dot) lesion distance (white bar) and each AI value, respectively. The touch‐up site is indicated by a dotted white circle (B), and the esophageal temperature alert is shown by the purple tags (C). An example case of no gap site in the left posterior segment (D). Left infero‐posterior segments showing four VisiTags and white and blue numbers indicating the center‐to‐center (black dot‐to‐black dot) lesion distance (white bar) and each AI value, respectively. AI min, minimum ablation index; ILD max, maximum interlesion distance; LI, left inferior; PA, postero‐anterior projection

We demonstrated the important role of AI min and ILD max values in predicting acute PVRs in complete segments. Furthermore, the lesion contiguity contributed to the prevention of acute PVRs in the left posterior segments with low AI min and in the other segments with high AI min. Therefore, the ILD max and AI min values complementarily aided each other in the left posterior segments, while AI min and ILD max were independently associated with acute PVRs in the other segments. Therefore, we should closely perform RFA only when we cannot apply sufficient RF energy due to increase in the temperature in the esophagus. Figure 4 shows examples of the VisiTags at the left posterior segments with and without acute PVRs (gaps or no gaps). It is understandable that the creation of a tightened lesion avoided the creation of gap, while a large lesion‐to‐lesion distance resulted in a gap creation even if the AI value was not sufficiently achieved. In contrast, in other segments, lesion contiguity was responsible for durable lesions, even if the AI min was high. This suggested that not only sufficient RF energy and CF but also lesion contiguity are required for the creation of durable lesions in other segments, such as the anterior ridge or carina, which have thicker walls. In the present study, the right posterior segments were also included in the other segments, and there were no significant differences in the ILD max between the segments with and without PVRs; therefore, the characteristics of the right posterior segments could not affect the characteristics of the entire posterior wall. The characteristics of the latter segments were based on those of the left posterior segments. Acute PVRs were more frequently observed in this study than in a previous study that used an AI‐guided procedure (13% segments vs 3% segments, respectively)^14^; however, the most prevalent sites where acute PVRs were observed were the left posterior sites in which AI value was low in the present study, presumably due to the extra attention paid to esophageal injury based on the temperature. Therefore, these results may aid in the creation of durable lesions, which could result in procedural improvements. Undoubtedly, performing RFA under the guidance of fixed AI values (>550 in the anterior and >400 in the posterior segments) yields excellent results; however, we considered that these fixed AI values were possibly excessive. Our results demonstrated that the median AI min value of 343 au and 258 au in the left supero‐ and infero‐posterior segments, respectively, were required to achieve acute success. In the other segments, there was no lesion where an AI min > 500 au was required to achieve acute success. These data suggest that further reduction of RF time and energy was possible.

This study has several limitations. First, this was a retrospective nonrandomized study and the patients who could not maintain sinus rhythm during the procedure were excluded; both these factors may have led to a selection bias. Second, a relatively small number of patients and segments of LA were included, possibly affecting the per‐segment analysis. Specifically, the region‐specific analysis shown in Table 2 was performed for 12 regions with 32 segments (for 32 patients) per region. Therefore, the analysis may have been statistically underpowered. Table 4 shows the findings of the region‐specific analysis performed for an extended area. Specifically, the left and right posterior regions each included 64 segments with 15 and nine acute PVRs, respectively. This may have increased the statistical power relative to that in the region‐specific analysis shown in Table 2. Interestingly, however, previous reports have also demonstrated regional differences in 3D electroanatomical mapping‐related indices, even though the incidence of acute PVRs was lower than that in the present study.^12^, ^14^ Third, we divided the AI min value into two groups according to median values, which was 297 in the left posterior segments and 412 in the other segments. We could not definitively determine whether such categorization was optimal and this may have potentially affected the study outcomes. Nevertheless, in the receiver‐operating characteristics curve analysis, AI min and ILD did not have a sufficient predictive value in detecting acute PVRs. Therefore, we adopted the median value for the categorization. Fourth, although the right‐sided epicardial connection via the carina has been recently reported,^15^ we did not treat the carina as a special site during categorization. Evaluation of acute PVRs, including carina segments, might provide an intriguing insight. Finally, we suggested that tight lesions could result in a reduction in acute PVRs in the left posterior segments; however, our data could not suggest a safety profile with respect to esophageal injury. In other words, from our findings, it remains unclear whether close tags were related to esophageal injury. This important aspect needs further investigation.

## 5 CONCLUSION

The AI min and ILD max are valuable indices in predicting acute PVRs. A shorter ILD may prevent acute PVRs when the AI min is low in the left posterior segments and high in the other segments, which suggests that a region‐oriented ablation is required for the creation of durable lesions.

## DISCLOSURE

The authors declare no conflict of interests for this article.

Clinical study IRB approval number/date: 4190‐R/October 3, 2019.

## Supporting information

Supplementary Material
