# Supplementary material for: Regional differences in the effects of the ablation index and interlesion distance on acute electrical reconnections after pulmonary vein isolation
Source: J Arrhythm. 2020 Jul 16;36(5):912–9. doi: 10.1002/joa3.12397 (PMC7532268; doi:10.1002/joa3.12397)
Supplement: Supplementary file 1 — Supplementary Material [file JOA3-36-912-s001.docx]

**
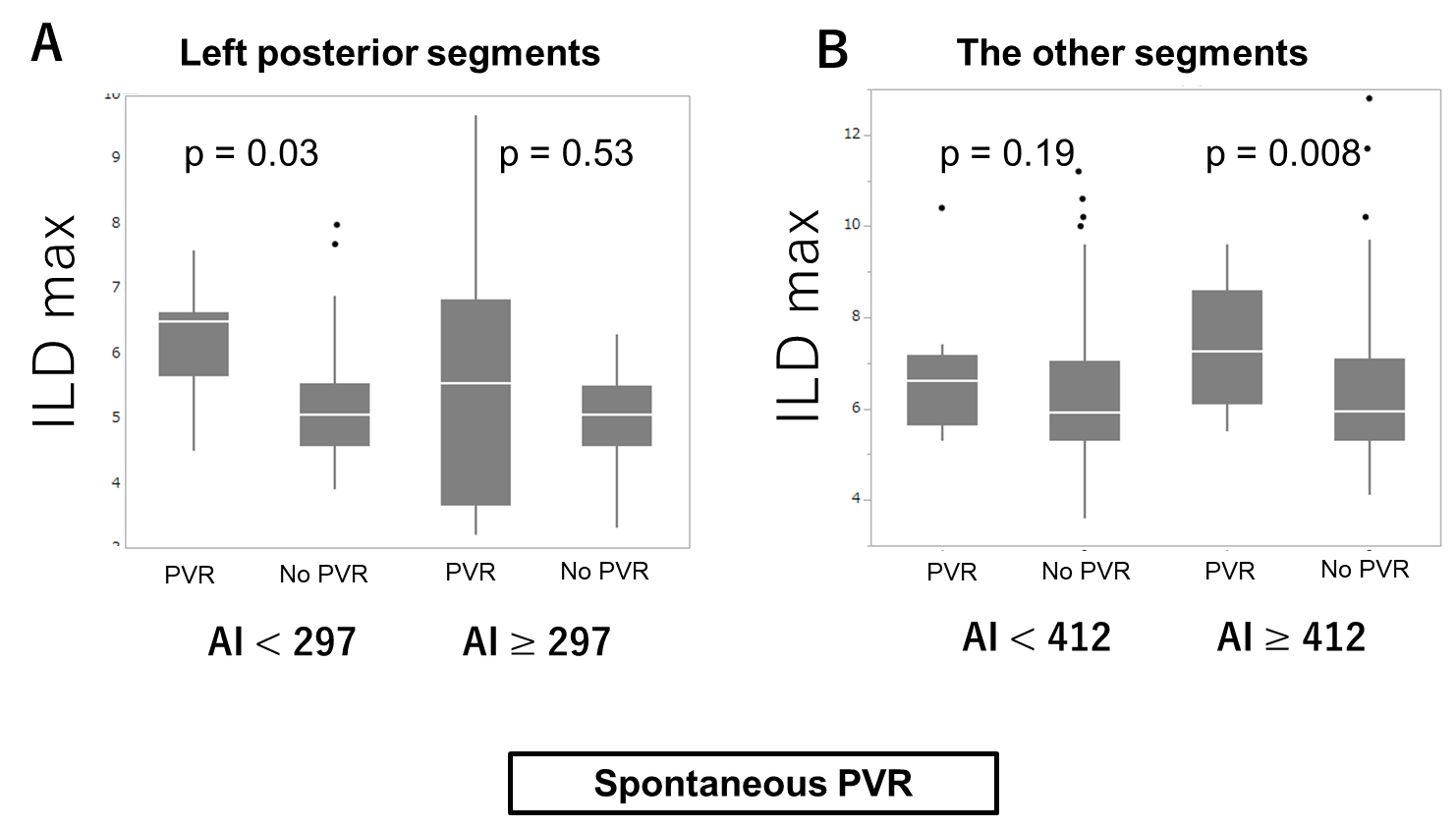
Supplementary Figure:** Panel A compares the maximum inter-lesion distance (ILD max) between the segments with spontaneous PVRs (not including dormant conduction) and the other segments according to the minimum ablation index (AI min) value in the left posterior segments; the segments with AI min either lower or higher than the median AI min are indicated on the left and right sides, respectively. Panel B illustrates the same for the other segments. AI min, minimum ablation index; PVR, pulmonary vein reconnection
